# Defining housekeeping genes suitable for RNA-seq analysis of the human allograft kidney biopsy tissue

**DOI:** 10.1186/s12920-019-0538-z

**Published:** 2019-06-17

**Authors:** Zijie Wang, Zili Lyu, Ling Pan, Gang Zeng, Parmjeet Randhawa

**Affiliations:** 1 Department of Urology, The First Affiliated Hospital of Nanjing Medical University, Nanjing, 210029 China; 2 Department of Pathology, The First Affiliated Hospital of Guangxi Medical University, Nanning, 530021 China; 3 Department of Nephrology, The First Affiliated Hospital of Guangxi Medical University, Nanning, 530021 China; 4 Department of Pathology, University of Pittsburgh Medical Center, E737 UPMC-Montefiore Hospital, 3459 Fifth Ave, Pittsburgh, PA 15213 USA

**Keywords:** RNA-sequencing, Kidney transplantation, Genes with housekeeping functions

## Abstract

**Background:**

RNA-seq is poised to play a major role in the management of kidney transplant patients. Rigorous definition of housekeeping genes (HKG) is essential for further progress in this field. Using single genes or a limited set HKG is inherently problematic since their expression might be altered by specific diseases in the patients being studied.

**Methods:**

To generate a HKG set specific for kidney transplantation, we performed RNA-sequencing from renal allograft biopsies collected in a variety of clinical settings. Various normalization methods were applied to identify transcripts that had a coefficient of variation of expression that was below the 2nd percentile across all samples, and the corresponding genes were designated as housekeeping genes. Comparison with transcriptomic data from the Gene Expression Omnibus (GEO) database, pathway analysis and molecular biological functions were utilized to validate the housekeeping genes set.

**Results:**

We have developed a bioinformatics solution to this problem by using nine different normalization methods to derive large HKG gene sets from a RNA-seq data set of 47,611 transcripts derived from 30 biopsies. These biopsies were collected in a variety of clinical settings, including normal function, acute rejection, interstitial nephritis, interstitial fibrosis/tubular atrophy and polyomavirus nephropathy. Transcripts with coefficient of variation below the 2nd percentile were designated as HKG, and validated by showing their virtual absence in diseased allograft derived transcriptomic data sets available in the GEO. Pathway analysis indicated a role for these genes in maintenance of cell morphology, pyrimidine metabolism, and intracellular protein signaling.

**Conclusions:**

Utilization of these objectively defined HKG data sets will guard against errors resulting from focusing on individual genes like 18S RNA, actin & tubulin, which do not maintain constant expression across the known spectrum of renal allograft pathology.

## Background

During the last decade, remarkable advances have been achieved in clinical medicine by the application of DNA microarray technology. Molecular signatures relevant to the diagnosis, prognosis and therapy have been discovered for numerous diseases [1–3]. In recent years, RNA-sequencing (RNA-seq) has been recognized as an attractive alternate technology for the same purpose. Compared to microarrays, RNA-seq provides a more comprehensive profiling of the transcriptome, with better quantitation, over a wider dynamic range, while allowing single base resolution, and detection of isoforms, RNA editing events, microRNAs and long noncoding RNAs [4–6]. The technology has been refined sufficiently to allow mRNA profiling of single cells. Challenges among the application of RNA-seq in clinical medicine include the need for an experimental design that includes sufficient numbers of biologic and technical replicates, and implementation of a mathematically valid bioinformatics pipeline to mine the large volume of data generated at a reasonable cost [7, 8].

The application of RNA-seq to the allograft kidney is at a very rudimentary stage. Rigorous definition of housekeeping genes (HKG) is essential for further progress in this field. HKG can be defined as genes ubiquitously expressed in all tissue compartments and cell-types regardless of their developmental stage, physiological condition and exposure to external stimuli [9]. HKG used in traditional clinical studies and classical biology experiments include 18S RNA, 28S RNA, tubulins, beta-actins, and glyceraldehyde-3-phosphate dehydrogenase (GAPDH). However, it is known that the expression of these genes is not constant through the cell cycle, and is further altered in response to injurious stimuli. Indeed 18 s RNA Is one of the biomarkers associated with acute rejection [10]. Actin is upregulated in chronic allograft dysfunction [11]. Tubulin is targeted by Colchicine, a drug used in patients with gout: it inhibits microtubule polymerization by binding to tubulin and block mitosis by acting as a ‘spindle poison’ [12]. These examples illustrate how use of single genes or a limited set HKG can be inherently problematic.

One potential solution to the problem is to use bioinformatics techniques and derive large HKG data sets for evaluation of high throughput gene expression data. This will ensure that alteration of a small number of genes due to experimental conditions does not unfavorably affect the overall data analysis. Accordingly, this study has developed HKG gene sets appropriate for assessment of differential gene expression using nine different nine normalization methods that include library size, total counts (TC), upper quartile (UQ), Median, Quantile, trimmed mean of M -values (TMM), reads per kilobase million (RPKM), transcripts per kilobase million (TPM) and DESeq. HKG lists are offered that are specific to particular normalization paradigms. In addition, there is a universal set of 42 housekeeping transcripts that are common to all nine individual analyses.

## Methods

### Clinical material

This study was approved by the University of Pittsburgh IRB (protocol # 10110393). Formalin fixed paraffin embedded renal allograft biopsies (*n* = 25) were derived from recipients diagnosed with acute tubular injury (ATI; *n* = 5), T cell-mediated rejection (TCMR; *n* = 5), interstitial fibrosis and tubular atrophy (IFTA; *n* = 5), and BK virus-associated nephropathy (BKVN; *n* = 5), as well as recipients with stable allograft function (STA; *n* = 5). Five native kidney biopsies with interstitial nephritis (ISN; *n* = 5) were also studied. The clinical and pathology parameters pertinent to these specimens have been published previously [13].

### RNA sequencing

RNA was extracted from 1 cubic mm pieces of formalin fixed paraffin embedded biopsy tissue using the Invitrogen PureLink™ FFPE RNA Isolation Kit (Catalog number: K156002), which includes a melting buffer to remove paraffin and a Proteinase K digestion step. cDNA libraries were constructed from 100 ng total RNA obtained using the Ion Ampliseq Transcriptome Human Gene Expression Kit from Life Technologies (Cat# A26325) and the manufacturers recommended protocol. Ampliseq Transcriptome analysis was performed by PrimBio Research Institute LLC, Exton, PA, USA, using an Ion Proton sequencer Ion Proton P1 chips, IonXpress barcodes, and Torrrent_Suite 5.0.4 software according to the manufacturer’s instructions. Briefly, Library Amp Primers were employed to amplify the purified cDNA library by PCR, and the yield and size of distribution of each library was run on Agilent 2100 Bioanalyzer. Approximately 100 pM of pooled barcoded libraries were used for templating using the Life Technologies Ion Chef Kit. Raw sequence files (fastq) were aligned to the human transcriptome (hg19) reference sequences in StrandNGS software. Gene and transcript annotations were retrieved from the Ensembl database to generate aligned SAM files, which were filtered on read quality (> 15), alignment score (≥90), match count (≤1) and mapping quality (≥25). RNA-seq quality control data on these biopsies has been published has been published [14]. RNA purity assessed by the A260/A280 ratio ranged from 1.87 to 2.0. RNA fragments of greater than 200 nucleotides in length comprised greater than 30% of the total RNA concentration. The mean sequence length in this RNA-Seq data set ranged from 66 to 117 nucleotides. Greater than 98.5% of the reads aligned to the human transcriptome with accuracy rates of greater than 97%. Our data has been submitted to the GEO database (GSE120495).

### Definition of HKG/normalization methods

The term HKG has been conceived to refer to genes responsible for maintenance of fundamental cellular function. These genes are ubiquitously expressed at approximately the same level in all cell-types regardless of developmental stage, physiological condition and presence of external stimuli [9, 15]. In this study, genes with expression coefficients of variance (CV) corresponding to the 2nd percentile across all 30 samples were assigned to the HKG category as has been suggested in the literature [16]. In a dataset of 47,613 genes this corresponded to 952 genes with the lowest CV. CV was calculated as the ratio of the standard deviation (SD) σ to the arithmetical mean μ of each gene.

Comparison of RNA-seq expression values across multiple samples requires normalization of data. Several normalization algorithms have been described in the literature, and we explored nine different methods, namely, library size, TC, UQ, Median, Quantile, RPKM, TPM, TMM and DESeq. Briefly, library size refers to the number of reads that aligned to the human genome. TC refers to transcript counts that remained after removing genes with an expression value of zero in all samples. The UQ scaling factor was calculated as a ratio of the 75th percentile of counts for each sample divided by the mean 75th percentile in all 30 samples [17]. The median scaling factor was obtained in the same manner using the 50th percentile [18]. Quantile normalization was implemented in R software using the “normalizeQuantiles()” function in the EBSeq package (Bioconductor version 3.6). This method sorts the test and reference distributions and proceeds to assign the highest value in latter to the highest value in the former [19]. The RPKM method attempts to normalize first for sequencing depth (per million reads) and then gene length (expressed in kilobases) [20]. TPM normalization proceeds in the reverse order: first, the raw read counts are divided by the length of the gene in kilobases, and then divided by the “per million” scaling factor [21]. TMM normalization was performed using the “calcNormFactors()” function in the edgeR package. The TMM method calculates a scaling factor based on a weighted trimmed mean of log gene expression ratios based on the assumption that most genes are not differentially expressed. Weights are assigned to account for the fact that genes with larger RNA-seq counts have lower variance, and data from both the upper and lower ends are trimmed prior to deriving a scaling factor for the sample library size [22]. Finally, DESeq normalization was implemented in DESeq package by calling the “estimateSizeFactors()” and “sizeFactors()” functions, which are also based on the hypothesis that most genes in the RNA-seq are not differentially expressed [23]. The performance of different normalization methods on our dataset was compared by calculating the bias and variance of genes in each HKG set [24]. The following formulae were used for the calculation of bias and variance, respectively:

$$ {\mathrm{Bias}}_{\mathrm{i}}=\kern0.5em \sqrt{\frac{1}{n}\kern0.5em {\Sigma}_{j=1}^n\kern1em {\left({\log}_2\left(\frac{ Ki j}{\overline{Ki.}}\right)\right)}^2} $$

$$ {\mathrm{Variance}}_{\mathrm{i}}=\frac{1}{n-1}{\sum}_{j=1}^n{\left({\log}_2\left(\frac{ Ki j}{\overline{Ki.}}\right)-{\log}_2{\left(\frac{ Ki j}{\overline{Ki.}}\right)}_i\right)}^2 $$

In these formulae, the K_ij_ represents the normalized read counts for *i*th gene from the *j*th sample, where the $\overline{Ki.}$ is the mean value of normalized read counts of each gene across 30 samples.

### Validation of HKG using published datasets

**Fig. 1:**
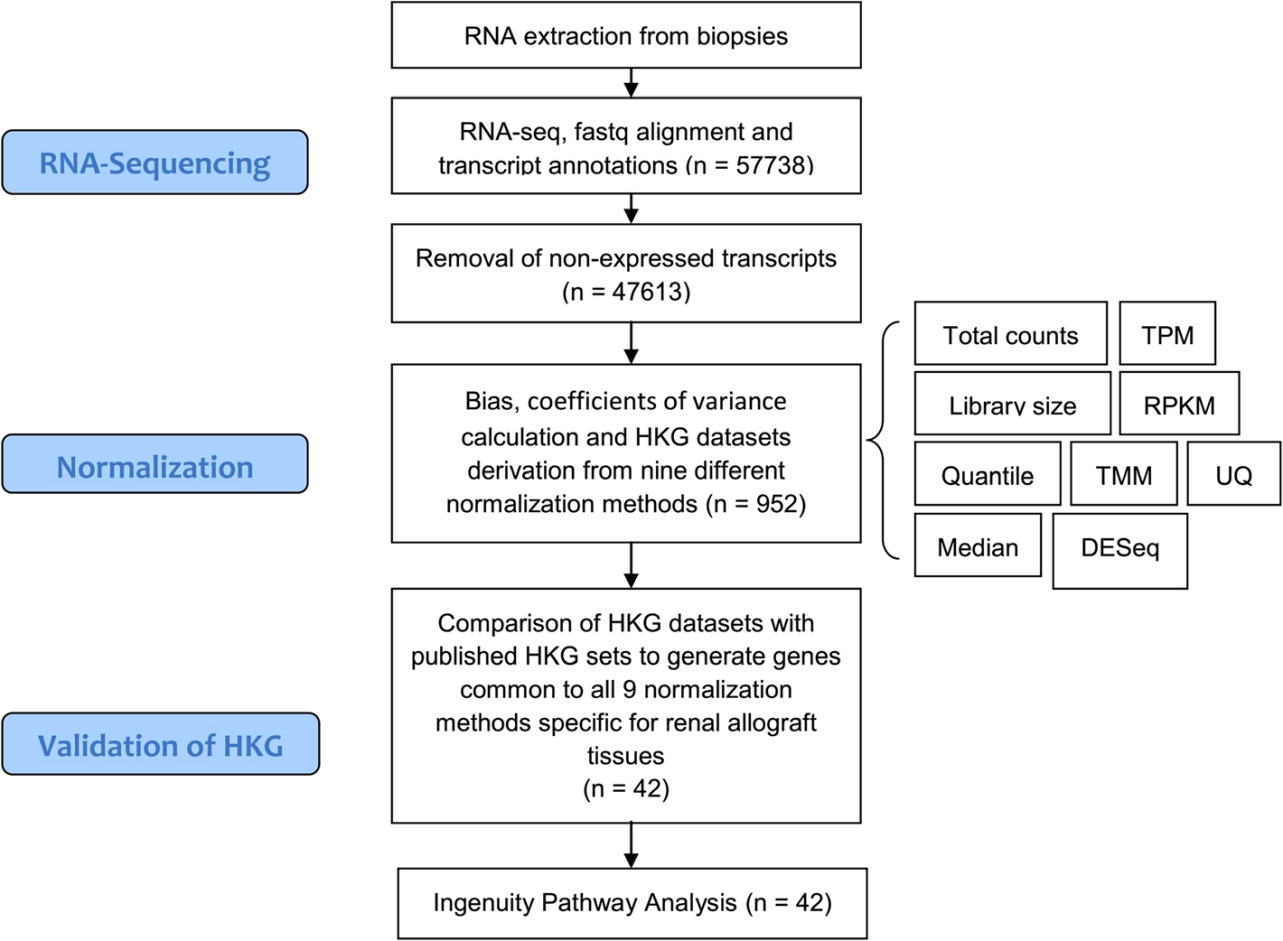
Flow diagram of the steps used to identify and validate HKG genes in this study

It was reasoned that genes classified HKG in this study would have minimal representation in lists of genes known to be differentially expressed in disease states that affect the kidney. Accordingly, we sought overlaps between the HKG dataset, and published gene sets derived from biopsy with T-cell mediated rejection, antibody mediated rejection, polyomavirus nephropathy, and chronic allograft damage [25–28]. Probe sets used to define disease associated genes in these studies were extracted from the NCBI GEO (Gene Expression Omnibus) database, and the corresponding gene and transcript annotations were obtained from the Ensembl database. Overlaps between gene lists of interest were defined by the “Compare” tool available in IPA® (Ingenuity Pathway Analysis) software (QIAGEN Biotechnology, Venlo, Netherlands). IPA core analysis was used to define the top-ranked canonical pathways and molecular functions associated with HKGs. A flow diagram of the steps used to identify and validate HKG in this study is presented as Fig. 1.

## Results

### Identification of housekeeping genes

**Fig. 2:**
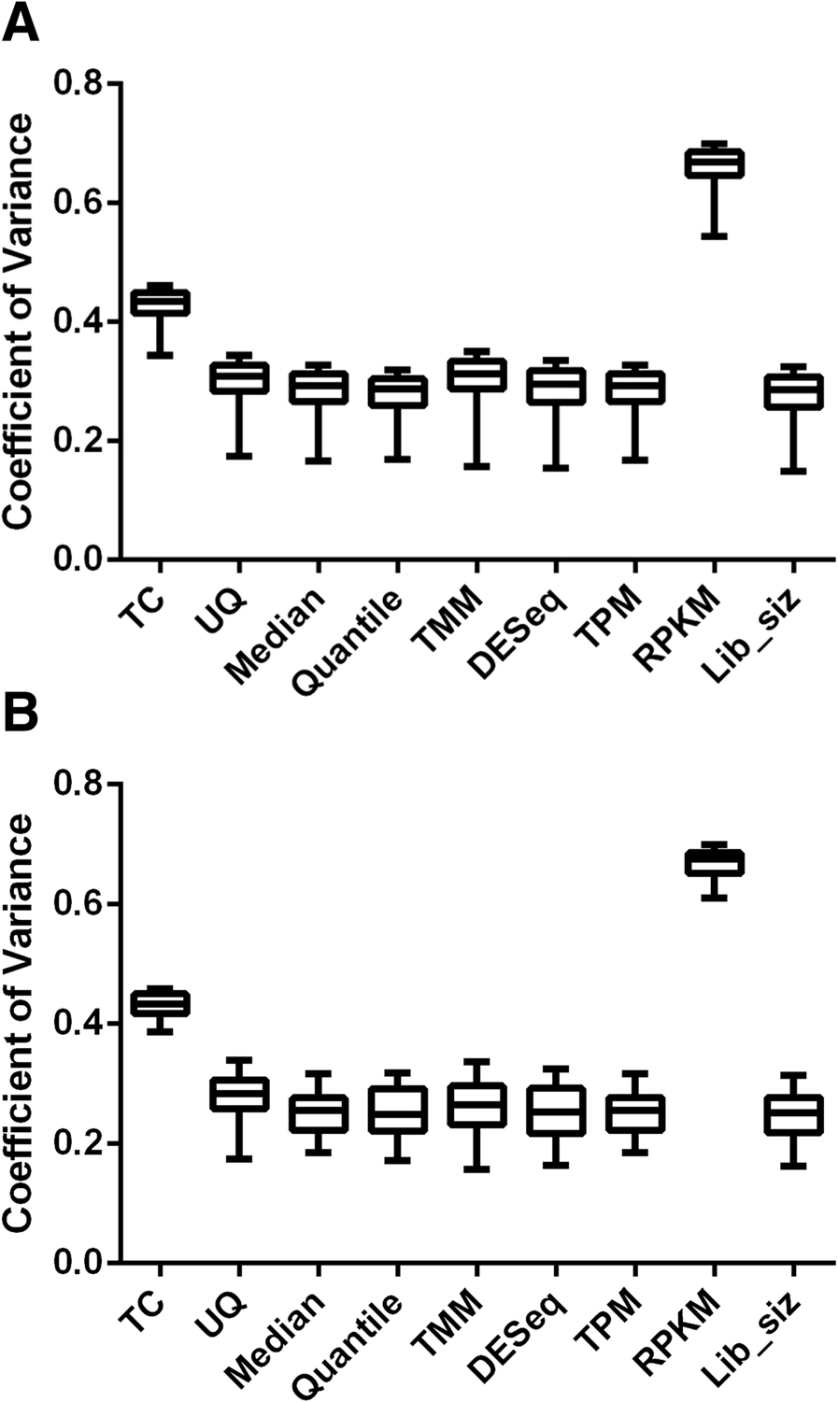
Box plots showing the median, first quartile, third quartile, and range of CV (coefficient of variance) for all 952 HKG defined by nine different normalization algorithms (**a**) and for the subset of 42 HKG common to all nine normalization methods (**b**) . **a** The median values (range) of CV in 952 HKGs defined by RPKM and TC are 0.67 (0.65–0.69) and 0.44 (0.41–0.45), respectively; whereas the mean values of CV defined by UQ, Median, Quantile, TMM, DESeq, TPM and Library size are 0.31 (0.28–0.33), 0.29 (0.27–0.31), 0.29 (0.26–0.31), 0.31 (0.29–0.33), 0.30 (0.27–0.32), 0.29 (0.27–0.31), 0.29 (0.26–0.31), respectively. **b** The median values (range) of CV in 42 common HKGs defined by RPKM and TC are 0.67 (0.65–0.69) and 0.43 (0.42–0.45), respectively; whereas the mean values of CV defined by UQ, Median, Quantile, TMM, DESeq, TPM and Library size are 0.28 (0.26–0.31), 0.25 (0.23–0.28), 0.25 (0.22–0.29), 0.26 (0.24–0.30), 0.25 (0.22–0.29), 0.25 (0.23–0.28), 0.25 (0.22–0.28), respectively. TC: total counts; UQ: upper quartile; TMM: trimmed mean of M-values; TPM: transcripts per kilobase million; RPKM: reads per kilobase per million mapped reads; e (see Materials and Methods section for details)

The mean number of reads with a quality score > Q30 obtained from the 30 biopsies ranged from 19 to 28 million, and yielded a total of 57,738 distinct reads that aligned to the hg19 human reference genome. After removing genes with an extracted expression value of zero in all biopsies, 47,613 transcripts remained for further consideration. Nine different HKG sets were created, one for each normalization method. Individual HKG expression accounted for only a small percentage of the total transcription activity in the samples. This is suggested by our calculation of expression ratios that represent mean normalized transcript counts of individual genes expressed as a proportion of the maximal transcript read count in the entire sample set. The numerical value of these expression ratios was less than < 0.05% for > 70% of the HKGs. (Table 1). The median coefficient of variation associated with most normalization methods was comparable (~ 0.3) except for the RPKM and TC methods where it was substantially higher (0.66 & 0.43 respectively) (Fig. 2a). The bias and variance of gene expression measurements was also the highest for these same two normalization methods (Table 1) indicating that the other methods tested by us provide much better data normalization. Similar results were obtained if CVs were calculated for the 42 HKG common to all normalization methods (Fig. 2b).

**Table 1:** Summary of HKG Datasets Defined in This Study Using 9 Different Normalization Methods

Normalization methods	Expression ratio*							Bias**	Variance**
	0–0.01 (%)	0.01–0.05 (%)	0.05–0.20 (%)	0.20–0.40 (%)	0.40–0.60 (%)	0.60–0.80 (%)	0.80–1.0 (%)		
TC	396 (41.60)	473 (49.68)	78 (8.19)	1 (0.11)	1 (0.11)	0 (0)	3 (0.32)	0.74	0.55
UQ	216 (22.69)	612 (64.29)	115 (12.08)	3 (0.32)	4 (0.42)	1 (0.11)	1 (0.11)	0.45	0.21
Median	157 (16.49)	643 (67.54)	142 (14.92)	7 (0.74)	2 (0.21)	0 (0)	1 (0.11)	0.45	0.22
Quantile	125 (13.13)	655 (68.80)	161 (16.91)	6 (0.63)	3 (0.32)	1 (0.11)	1 (0.11)	0.42	0.18
TMM	236 (24.79)	599 (62.92)	108 (11.34)	4 (0.42)	4 (0.42)	0 (0)	1 (0.11)	0.47	0.23
DESeq	231 (24.26)	610 (64.08)	104 (10.89)	4 (0.42)	2 (0.21)	0 (0)	1 (0.11)	0.43	0.19
TPM	157 (16.49)	643 (67.54)	142 (14.92)	7 (0.74)	2 (0.21)	0 (0)	1 (0.11)	0.45	0.22
RPKM	603 (63.34)	319 (33.51)	26 (2.73)	2 (0.21)	0 (0)	0 (0)	2 (0.21)	1.04	1.03
Lib_size	202 (21.22)	617 (64.81)	123 (12.92)	7 (0.74)	2 (0.21)	0 (0)	1 (0.11)	0.43	0.20

Abbreviations: *TC* total counts, *UQ* upper quantile, *TMM* trimmed mean of M-values, *DESeq* a differential expression package implemented in R, *TPM* transcripts per kilobase million, *RPKM* reads per kilobase per million mapped reads, *Lib_size* library size

*The expression ratio of each housekeeping gene was calculated by its mean normalized read divided by the maximum reads in its corresponding HKG set

**The bias and variance of each normalization method was calculated by the formulae

### Validation of housekeeping genes

By definition, HKG maintain basic cellular functions and their expression does should not change in different disease states. This prediction was verified using public datasets for 4 common pathologic conditions in the kidney, namely, TCMR, antibody mediated rejection (ABMR), BKVN and chronic allograft injury manifesting as IFTA (Table 2). None of the 952 genes identified as HKG in this study were differentially expressed in human allograft biopsies with TCMR. For the gene lists associated with the remaining biopsy-diagnoses, an overlap of no more than 3 genes was seen with our HKG lists. This is remarkable since Gene Expression Omnibus data used in these comparisons was derived from more than 1000 biopsies.

**Table 2:** Overlaps^a^ Between Gene Expression Datasets Derived from Diseased Allograft Kidney & HKG Defined in This Study

Reference	#Biopsies	#of DE transcripts	Biopsy Diagnosis	Normalization method Used to Define HKG								
				TC (%)	UQ (%)	Median (%)	Quantile (%)	TMM (%)	DESeq (%)	TPM (%)	RPKM (%)	Lib_siz (%)
[25]	703	453	ABMR	2(0.40)	2(0.44)	2(0.44)	2(0.44)	1(0.22)	2(0.44)	2(0.44)	8(1.77)	1(0.22)
[26]	708	82	TCMR	0(0)	0(0)	0(0)	0(0)	0(0)	0(0)	0(0)	0(0)	0(0)
[27]	168	206	BKVN	3(1.46)	5(2.43)	3(1.46)	3(1.46)	3(1.46)	3(1.46)	3(1.46)	3(1.46)	3(1.46)
[28]	204	82	Chronic allograft damage	1(1.22)	1(1.22)	2(2.44)	2(2.44)	1(1.22)	1(1.22)	2(2.44)	1(1.22)	2(2.44)

Abbreviations: *ABMR* antibody mediated rejection, *DE* differentially expressed, *TCMR* T-cell mediated rejection, *BKVN* polyomavirus nephropathy, For other abbreviations, see legend to Table 1

^a^The total number of overlapping genes with the specified datasets is enumerated

As an alternate approach to validating the HKG datasets obtained in this study, we compared the constituent genes with HKG lists defined by other investigators using varied technical approaches including expressed sequence tags, DNA microarray, RNA-seq, and massively parallel signature sequencing (Table 3) [29–35]. HKG derived from sequencing based technologies gave the largest number of genes (279 to 656) in common with our own RNA-seq derived gene list. There were fewer (80 to 117) genes shared with microarray technology-based lists. It is apparent that HKG gene identification can be affected by both the normalization method used as well as the technology applied to measure gene expression. The type of tissue analyzed is also an important variable. Whereas all our samples represent the allograft kidney, the aforementioned prior studies included multiple organs in their analysis. Thus, different HKG gene sets can be equally valid depending on the clinical setting and sample set being investigated.

**Table 3:** Comparison of Published housekeeping genes with HKG Datasets Defined in This Study

Study	#samples	#HKG	#Tissues/cells studied	Technique	Normalization method	Housekeeping Gene Set Stratified by Normalization Method^a^								
						TC (%)	UQ (%)	Median (%)	Quantile (%)	TMM (%)	DESeq (%)	TPM (%)	RPKM (%)	Lib_siz (%)
(4)	142	1789	79	Microarray	NA^b^	94 (5.25)	91 (5.09)	115 (6.43)	111 (6.20)	92 (5.14)	89 (4.97)	115 (6.43)	76 (4.25)	96 (5.37)
(5)	18	2403	18	Microarray	NA	110 (4.58)	145 (6.03)	158 (6.58)	161 (6.70)	132 (5.49)	124 (5.16)	158 (6.58)	103 (4.29)	133 (5.53)
(6)	42	1522	42	Microarray	NA	89 (5.84)	112 (7.36)	117 (7.69)	115 (7.56)	87 (5.72)	88 (5.78)	117 (7.69)	80 (5.26)	92 (6.04)
(6)	NA	15,050	32	sequencing_MPSS	TPM	516 (3.43)	578 (3.84)	566 (3.76)	581 (3.86)	550 (3.65)	559 (3.71)	566 (3.76)	489 (3.25)	559 (3.71)
(7)	2502	6909	18	Sequencing_EST	NA	398 (5.76)	542 (7.84)	533 (7.71)	551 (7.98)	463 (6.70)	458 (6.63)	533 (7.71)	369 (5.34)	471 (6.82)
(8)	NA	12,714	19	sequencing_EST	NA	583 (4.59)	627 (4.93)	642 (5.05)	656 (5.16)	610 (4.80)	620 (4.88)	642 (5.05)	546 (4.29)	628 (4.94)
(9)	NA	7896	32	RNA-Seq	RPKM	514 (6.51)	628 (7.95)	654 (8.28)	656 (8.31)	601 (7.61)	594 (7.52)	654 (8.28)	441 (5.59)	615 (7.79)
(10)	16	3804	16	RNA-Seq	RPKM	279 (7.33)	361 (9.49)	372 (9.78)	379 (9.96)	317 (8.33)	315 (8.28)	372 (9.78)	212 (5.57)	329 (8.64)

Abbreviations: *EST* expressed sequence tags, *HKG* housekeeping genes, *MPSS* Massively parallel signature sequencing, *ABMR* antibody mediated rejection, *DE* differentially expressed, *TCMR* T-cell mediated rejection, *PVAN* polyomavirus nephropathy, *NA* not available; for other abbreviations, see legend to Table 1

^a^The total number (%) of overlapping genes with the specified datasets is enumerated. Percentage calculations are based on the total number of HKG in column 3

^b^The normalization methods in these references were not mentioned, but the most common method used for microarray data is Quantile normalization

### Pathway analyses

**Fig. 3:**
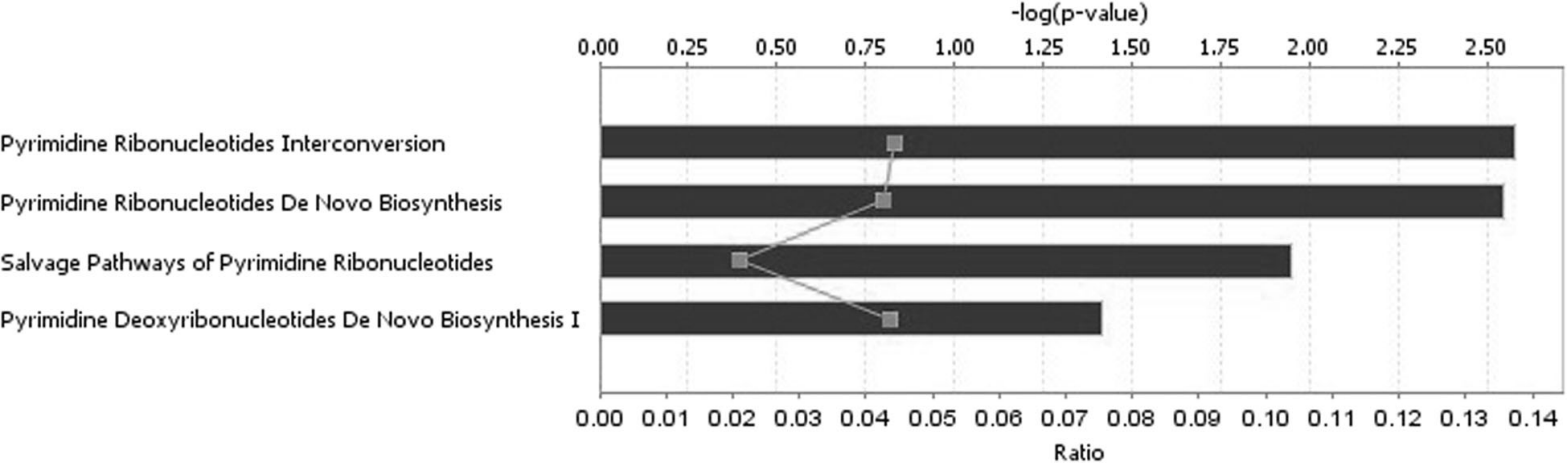
Canonical pathways identified by IPA core analysis as over-represented amongst 42 HKG common to 9 different data normalization methods. Pathways meeting statistical confidence thresholds preset in IPA are identified on the Y-axis (−log_10_ *p* = 1.3, right-tailed Fisher’s exact test). The lower X-axis and the line diagram display the proportion of total genes in the specified pathway that meet the cutoff criteria for identification

**Fig. 4:**
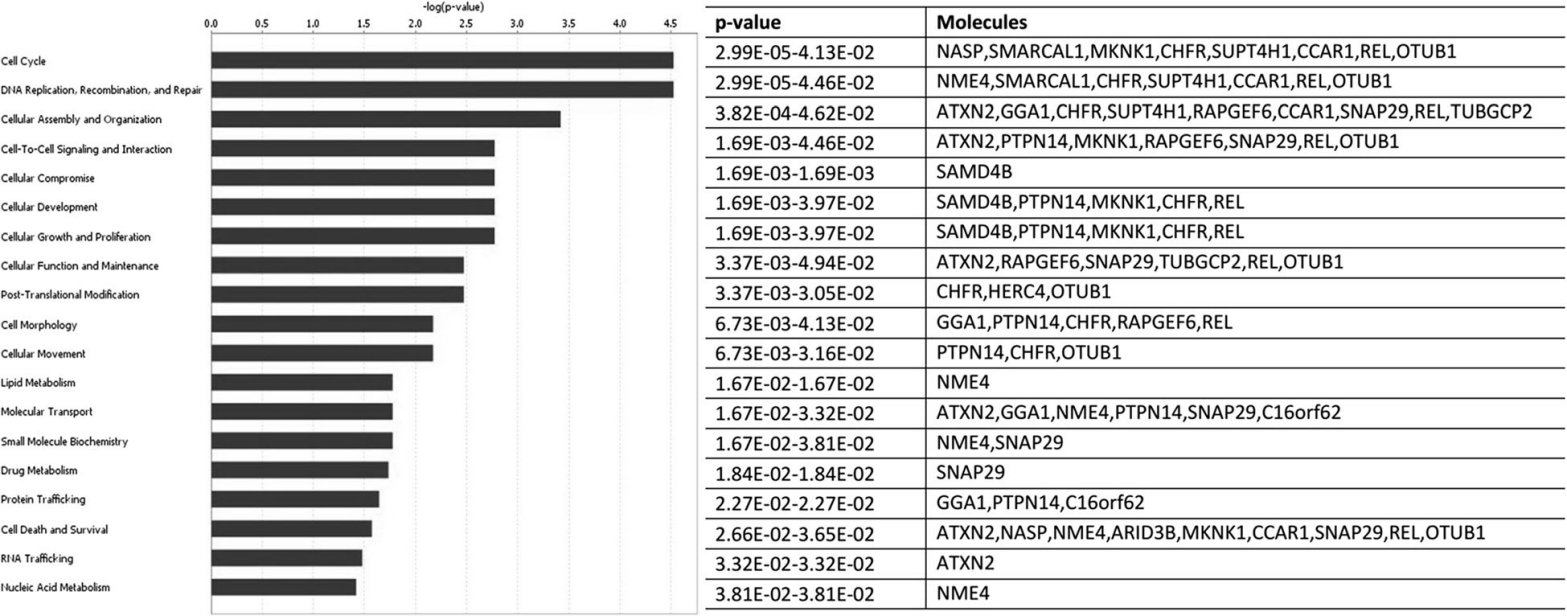
Top 20 physiologic functions associated with 42 HKG common to all biopsies and normalization methods. Physiological functions meeting statistical confidence thresholds (−log_10_ p = 1.3, right-tailed Fisher’s exact test)

Ingenuity pathway analysis was performed on 42 genes common to 9 HKG sets derived from different normalization methods. The Entrez gene names and molecular functions of these genes are listed in Table 4. The majority are involved in chromatin, core promoter, DNA, mRNA, protein, or ATP binding, while others represent ubiquitous enzymes belonging to the protein kinase, phosphatase, protease, ligase, ATPase or GTPase family. Physiologic functions mediated by these housekeeping genes included regulation of the cell cycle, cell to cell signaling, post-translational modifications, cell morphology, cell movement, molecular transport, and lipid or nucleic acid metabolism (Figs. 3 and 4, Table 5). The top 4 canonical pathways identified all involved de novo or salvage pathways of pyrimidine biosynthesis, including pyrimidine ribonucleotides interconversion, pyrimidine ribonucleotides de novo biosynthesis, and pyrimidine deoxyribonucleotides de novo biosynthesis. Notably, less than 5% of the genes in these pathways met the criterion for being classified as a housekeeping gene. The majority of the remaining canonical pathways were related to protein signaling mediated by the protein kinase A, p38 MAPK, RhoA, CREB, ERK/MAPK, Eif4, p70S6K, IL-12, glucocorticoid receptor, estrogen receptor, or progesterone receptor pathways.

**Table 4:** Housekeeping Genes (*n* = 42) Common to All Normalization Methods

Entrez Gene ID	Transcripts	Entrez Gene Name	Molecular function
51,433	ANAPC5	anaphase promoting complex subunit 5	protein phosphatase binding
25,906	ANAPC15	anaphase promoting complex subunit 15	anaphase-promoting complex
10,620	ARID3B	AT-rich interaction domain 3B	transcription regulator
285,598	ARL10	ADP ribosylation factor like GTPase 10	small GTPase mediated signal transduction
6311	ATXN2	ataxin 2	epidermal growth factor receptor binding
57,020	C16orf62	chromosome 16 open reading frame 62	protein binding
132,200	C3orf49	chromosome 3 open reading frame 49	unknown
55,749	CCAR1	cell division cycle and apoptosis regulator 1	core promoter binding
202,243	CCDC125	coiled-coil domain containing 125	regulation of cell motility
60,492	CCDC90B	coiled-coil domain containing 90B	protein binding
55,743	CHFR	checkpoint with forkhead and ring finger domains	E3 ubiquitin-protein ligase
207,063	DHRSX	dehydrogenase/reductase X-linked	oxidoreductase activity
83,786	FRMD8	FERM domain containing 8	protein binding
26,088	GGA1	golgi associated, gamma adaptin ear containing, ARF binding protein 1	cellular protein metabolic process
26,091	HERC4	HECT and RLD domain containing E3 ubiquitin protein ligase 4	transferase activity; ubiquitin-protein ligase activity
8569	MKNK1	MAP kinase interacting serine/threonine kinase 1	ATP binding; calcium-dependent protein serine/threonine kinase activity
4678	NASP	nuclear autoantigenic sperm protein	histone binding; Hsp90 protein binding;
4833	NME4	NME/NM23 nucleoside diphosphate kinase 4	ubiquitous enzymes
55,611	OTUB1	OTU deubiquitinase, ubiquitin aldehyde binding 1	NEDD8-specific protease activity
11,243|100,527,963	PMF1/PMF1-BGLAP	polyamine modulated factor 1	leucine zipper domain binding
5431	POLR2B	RNA polymerase II subunit B	chromatin binding
11,128	POLR3A	RNA polymerase III subunit A	chromatin binding
84,197	POMK	protein-O-mannose kinase	ATP binding; carbohydrate kinase activity
379,025	PSMA3-AS1	PSMA3 antisense RNA 1	unknown
5784	PTPN14	protein tyrosine phosphatase, non-receptor type 14	hydrolase activity; phosphatase activity
51,735	RAPGEF6	Rap guanine nucleotide exchange factor 6	GTP-dependent protein binding
5966	REL	REL proto-oncogene, NF-kB subunit	chromatin binding; DNA binding
8568	RRP1	ribosomal RNA processing 1	RNA binding
146,923	RUNDC1	RUN domain containing 1	GTPase activator activity; Rab GTPase binding
55,095	SAMD4B	sterile alpha motif domain containing 4B	mRNA binding;
22,950	SLC4A1AP	solute carrier family 4 member 1 adaptor protein	mRNA binding; protein binding
7871	SLMAP	sarcolemma associated protein	protein binding
50,485	SMARCAL1	SWI/SNF related, matrix associated, actin dependent regulator of chromatin, subfamily a like 1	ATP binding; DNA-dependent ATPase activity
9342	SNAP29	synaptosome associated protein 29	protein binding; SNAP receptor activity
23,020	SNRNP200	small nuclear ribonucleoprotein U5 subunit 200	ATP binding; ATP-dependent helicase activity
6827	SUPT4H1	SPT4 homolog, DSIF elongation factor subunit	metal ion binding; protein binding
25,771	TBC1D22A	TBC1 domain family member 22A	14–3-3 protein binding; GTPase activator activity
440,944	THUMPD3-AS1	THUMPD3 antisense RNA 1	unknown
100,506,779	TSPOAP1-AS1	TSPOAP1 antisense RNA 1	unknown
10,844	TUBGCP2	tubulin gamma complex associated protein 2	gamma-tubulin binding
23,038	WDTC1	WD and tetratricopeptide repeats 1	enzyme inhibitor activity
27,300	ZNF544	zinc finger protein 544	DNA binding; metal ion binding

**Table 5:** Canonical Pathways identified by IPA software for 42 Housekeeping Genes Common to All Normalization Methods

Ingenuity Canonical Pathways	-log(*p*-value)	Ratio	Molecules
Pyrimidine Ribonucleotides Interconversion	2.58	0.0444	NME4,SMARCAL1
Pyrimidine Ribonucleotides De Novo Biosynthesis	2.55	0.0426	NME4,SMARCAL1
Salvage Pathways of Pyrimidine Ribonucleotides	1.95	0.0211	NME4,POMK
Pyrimidine Deoxyribonucleotides De Novo Biosynthesis I	1.42	0.0435	NME4
Nucleotide Excision Repair Pathway	1.24	0.0286	POLR2B
Assembly of RNA Polymerase II Complex	1.09	0.02	POLR2B
Pyridoxal 5′-phosphate Salvage Pathway	0.983	0.0154	POMK
Mitotic Roles of Polo-Like Kinase	0.977	0.0152	ANAPC5
Protein Kinase A Signaling	0.836	0.005	PTPN14,ANAPC5
Androgen Signaling	0.767	0.00901	POLR2B
p38 MAPK Signaling	0.736	0.00833	MKNK1
RhoA Signaling	0.723	0.00806	RAPGEF6
Estrogen Receptor Signaling	0.711	0.00781	POLR2B
Hereditary Breast Cancer Signaling	0.665	0.00694	POLR2B
IL-12 Signaling and Production in Macrophages	0.66	0.00685	REL
Regulation of eIF4 and p70S6K Signaling	0.632	0.00637	MKNK1
CREB Signaling in Neurons	0.568	0.00538	POLR2B
RAR Activation	0.56	0.00526	REL
ERK/MAPK Signaling	0.541	0.005	MKNK1
Systemic Lupus Erythematosus Signaling	0.499	0.00444	SNRNP200
Huntington’s Disease Signaling	0.461	0.004	POLR2B
Protein Ubiquitination Pathway	0.441	0.00377	ANAPC5
Glucocorticoid Receptor Signaling	0.358	0.00295	POLR2B
Axonal Guidance Signaling	0.27	0.00221	MKNK1

## Discussion

The primary purpose of this study was to identify HKG appropriate for analyzing RNA-seq data derived from human renal allograft biopsies. It is expected that RNA-seq technology will be increasingly applied to discover molecular signatures relevant to the diagnosis, prognosis and therapy of diseases that commonly afflict kidney transplant recipients. The work performed has identified 9 HKG sets using different normalization methods and the question arises which gene set is most applicable to the analysis of gene expression data derived from renal allograft biopsies. Zyprych-Walczak et al. [36] analyzed transcripts from mammary epithelial cell lines, B-cells, and blood or bone marrow samples from patients with acute myeloid leukemia. They compared six normalization algorithms with respect to sensitivity, specificity, classification errors, and generation of diagnostic plots, and found that bias and variance were appropriate indices to compare the performance of different normalization methods. Application of this principle to our data indicates normalization using only the TC or RPKM methods is not advisable. The other normalization methods give essentially comparable results, although the quantiles method is nominally better than all the others that were tested. The basic idea behind RPKM is to normalize the reads first by total reads and then by gene length. Previous studies have confirmed the suboptimal performance of this method [17, 37, 38]. Interestingly, better performance was seen with TPM which differs from RPKM only in that normalization for gene length precedes correction for total reads. This reversal in the order of operations led to relatively uniform transcript counts in all 30 biopsies. However, in one prior study both TC and RPKM led to unsatisfactory results [39]. Two prior investigations noted that the quantile method is associated with lower variance in observed gene expression data, but there is a tradeoff that results in the introduction of some bias [19, 40]. Another study reported that DESeq method is the best for the normalization [39].

Our assessment of the published literature is that no single normalization method can be universally recommended for all data sets. HKG lists vary depending on study design, tissues analyzed, sequencing technology, normalization methods, as well as criteria and tools for housekeeping gene selection [41, 42]. Data distribution and analytic plans can influence the choice of normalization method: e.g. if most genes have low expression, upper quantile rather than median normalization should be preferred. On the other hand, if differential expression is to be performed by the DESeq program, the normalization algorithm incorporated in the software can work directly with unnormalized RNA-seq counts. Finally, we suggest that when working with renal allograft biopsies, the problem of choosing the right HKG set can be circumvented by using the list of 42 genes (Table 4) that is common to gene sets derived by 9 different algorithms.

The HKG proposed in this study have been validated with reference to publicly available external gene expression datasets obtained on an independent platform, namely, the Affymetrix DNA microarray analysis system. These latter datasets were derived from kidney transplant biopsies with TCMR, ABMR, BKVN or i-IFTA. A second observation that validates our HKG gene lists is that these share up to 656 genes with other RNA-seq derived gene lists in the literature. Finally, our IPA analysis is consistent with the proposed housekeeping function of these genes, and is concordant with putative cellular and biologic functions of other HKG reported in the literature. These reported functions include RNA processing, RNA splicing, DNA repair and mRNA metabolic processes [43], cell morphology and signaling, defense/apoptosis, ribosomal protein signaling/communication, structure/motility [44, 45], and biogenesis of nucleotides/amino acids and protein localization [35]. It is to be noted that some genes such as GAPHD and beta actin (ACTB), which are widely used in biological experiments as housekeeping controls, do not appear in our HKG set [46–48]. Likewise, our gene list does not include 8 genes that have been suggested to be suitable as a reference set for studies of the non-transplanted kidney [49].

## Conclusion

In summary, we have developed several different HKG gene lists applicable to RNA-seq data derived from for human allograft kidney biopsies and processed by a variety of normalization methods. We have also assembled a universal set of 42 HKG that can be used without regard to the actual normalization procedure used. The study is limited by the small number of biopsies studied and use of formalin fixed paraffin embedded tissue, which may not be optimal to detect genes expressed at low abundance. However, low abundance genes have high variance and are not good candidates for the HKG designation. Importantly, the general bioinformatics approach that we have outlined is applicable to define HKG for RNA-seq datasets of any size and RNA quality for transplantation of all organs. Appropriate normalization of samples with a comprehensive set of HKG provides a mechanism to correct for batch effects, which can be a significant obstacle in the implementation of RNA-seq as a monitoring tool in the transplant clinic.

## Data Availability

The datasets supporting the conclusions of this article are available in the Gene Expression Omnibus (GEO) database (GSE120495, https://www.ncbi.nlm.nih.gov/geo/).

## Abbreviations

ABMR: Antibody mediated rejection
ACTB: Beta actin
ATI: Acute tubular injury
CV: Coefficients of variance
GAPDH: Glyceraldehyde-3-phosphate dehydrogenase
GEO: Gene Expression Omnibus
HKG: Housekeeping genes
IFTA: Interstitial fibrosis and tubular atrophy
IPA: Ingenuity Pathway Analysis
ISN: Interstitial nephritis
RNA-seq: RNA-sequencing
RPKM: Reads per kilobase million
STA: Stable allograft function
TC: Total counts
TCMR: T cell-mediated rejection
TMM: Trimmed mean of M -values
TPM: Transcripts per kilobase million
UQ: Upper quartile
